# Does Sedentary Behavior Predict Academic Performance in Adolescents or the Other Way Round? A Longitudinal Path Analysis

**DOI:** 10.1371/journal.pone.0153272

**Published:** 2016-04-07

**Authors:** Jorge Lizandra, José Devís-Devís, Esther Pérez-Gimeno, Alexandra Valencia-Peris, Carmen Peiró-Velert

**Affiliations:** 1 Departament d’Educació Física i Esportiva, Universitat de València, Valencia, Spain; 2 Departament de Didàctica de l’Expressió Musical, Plàstica i Corporal, Universitat de València, Valencia, Spain; University of Tennessee Health Science Center, UNITED STATES

## Abstract

This study examined whether adolescents’ time spent on sedentary behaviors (academic, technological-based and social-based activities) was a better predictor of academic performance than the reverse. A cohort of 755 adolescents participated in a three-year period study. Structural Equation Modeling techniques were used to test plausible causal hypotheses. Four competing models were analyzed to determine which model best fitted the data. The Best Model was separately tested by gender. The Best Model showed that academic performance was a better predictor of sedentary behaviors than the other way round. It also indicated that students who obtained excellent academic results were more likely to succeed academically three years later. Moreover, adolescents who spent more time in the three different types of sedentary behaviors were more likely to engage longer in those sedentary behaviors after the three-year period. The better the adolescents performed academically, the less time they devoted to social-based activities and more to academic activities. An inverse relationship emerged between time dedicated to technological-based activities and academic sedentary activities. A moderating auto-regressive effect by gender indicated that boys were more likely to spend more time on technological-based activities three years later than girls. To conclude, previous academic performance predicts better sedentary behaviors three years later than the reverse. The positive longitudinal auto-regressive effects on the four variables under study reinforce the ‘success breeds success’ hypothesis, with academic performance and social-based activities emerging as the strongest ones. Technological-based activities showed a moderating effect by gender and a negative longitudinal association with academic activities that supports a displacement hypothesis. Other longitudinal and covariate effects reflect the complex relationships among sedentary behaviors and academic performance and the need to explore these relationships in depth. Theoretical and practical implications for school health are outlined.

## Introduction

During the last decade, a growing concern has arisen regarding physical and social health consequences of a sedentary lifestyle among adolescents. Excessive time spent on screen-related sedentary behaviors is associated with energy intake and obesity [1], and metabolic and cardiovascular disease, especially among girls [2]. Increased levels of electronic media use are also related with children and adolescents’ poor health status, health-related quality of life and well-being outcomes, being more important in boys than girls [3, 4]. Positive relationships are observed with sleep problems and insufficient sleep time [5], illicit drug use and low self-esteem [6], and quality in peer relationships [7] in both, boys and girls.

Some research has also suggested that time engaged in screen-related sedentary behaviors negatively affects children and adolescents’ academic performance (a group of cognitive skills, attitudes, academic behaviors and achievement) [8, 9]. Moreover, TV, videogame and computer use among adolescents, especially boys, when extended two hours of consumption per day, interfere with studying and reading books [10, 11]. Different types of screen media use, including mobile phone usage, were significantly related to high ratings of distractibility for academic tasks [12, 13]. School grades have also been affected by TV viewing, videotapes and overall screen-related sedentary time in more boys than girls [7, 14].

Nevertheless, other studies found positive consequences of screen-related sedentary behaviors on adolescents’ academic performance. For instance, internet use was positively related to reading skills and school performance [15] and playing games contributed to developing some cognitive skills among children and adolescents [16]. Playing computer games may produce improvements in reaction times, attentional skills and changes through pro-social behavior [17]. Furthermore, emailing, gathering information or doing homework on the computer contributes positively to academic performance and, used in moderation, to adolescents’ social development and well-being in boys and girls [18, 19].

These contradictory results about the relationships among screen-related sedentary usage and academic performance suggest the need for further in-depth research on such linkages.

Despite the research evidence on prediction and causal effects of academic performance on different adolescent health behaviors [20–25], a remarkable absence of evidence is observed on the impact that academic performance may have on different sedentary behaviors. To the best of our knowledge, academic performance shows a positive effect on self-esteem, personal control and prosocial behavior among school adolescents, as well as adult employment and income, and better future health [20–22]. It also shows a negative association in later delinquency, violent behavior, substance use and adult allostatic load [23–25]. Nevertheless, the lack of evidence on subsequent sedentary behaviors is striking, especially when academic achievement is considered to contribute to life course health [21].

Against this backdrop, one may wonder whether there is empirical support for prediction and preponderance of academic performance over screen-related sedentary usage or even if there are reciprocal effects. Therefore, the first purpose of the current study is to test, via a longitudinal path analysis, four competing models that relate different sedentary behaviors (academic, technological and social leisure) and academic performance in order to determine which is the best predictor of the other. According to the previous literature review, gender may affect the relationships between sedentary behaviors and academic performance. Thus, a second purpose emerges to assess the potential moderating effects of gender on these relationships.

## Materials and Methods

### Ethics Statement

Materials and procedures were approved by the individual schools, the school districts and the Ethics Committee of the Universitat de València. Written informed consent forms were obtained from parents and from participants aged 18 years or over.

### Design and Participants

**Fig 1:**
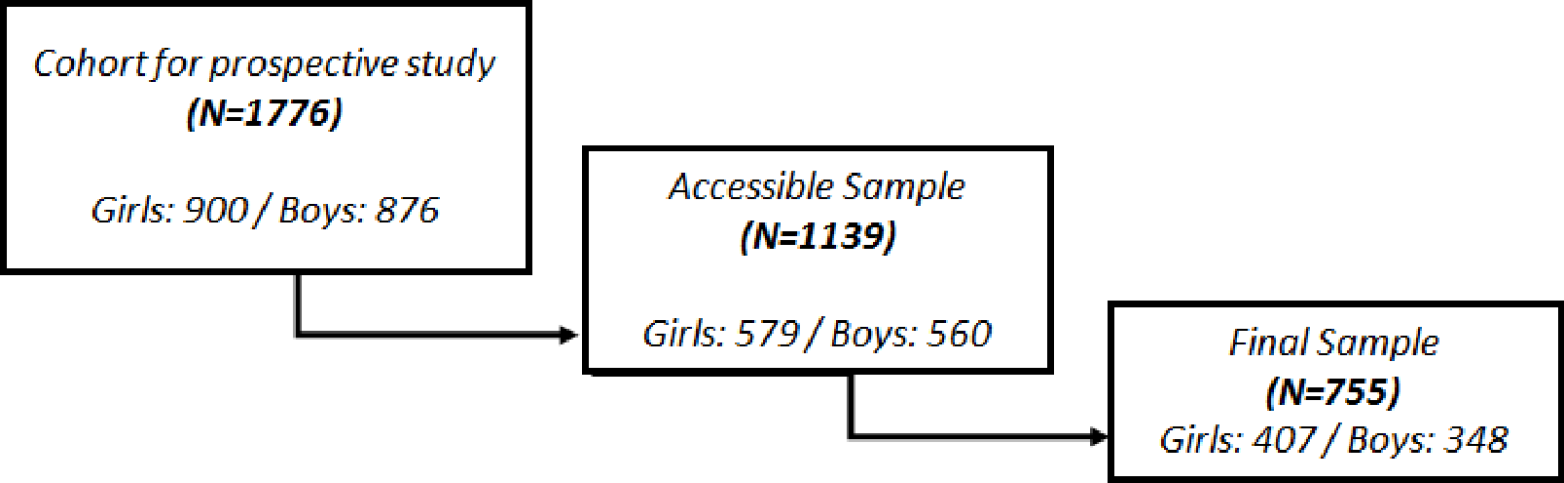
Key stages in the process for obtaining the final sample of the study

A prospective cohort study was designed to examine the use that school adolescents made of sedentary activities and their academic performance. Participants belonged to 13 secondary schools (7 state and 6 private) from six geographical areas that include the whole Spain (North, South, Center, North-East, East and Canary Islands). These areas were employed in prior studies [14, 26]. From a previous cross-sectional representative study [14], developed with 3,095 students in Fall 2010, the cohort for this study was defined by those students who were at that time in grades 7, 8 and 9 (Wave I) and could still be enrolled in these schools three years later, in grades 10, 11 and 12 (Wave II). From the original cohort of 1776 students belonging to grades 7, 8 and 9 from all schools participating in Wave I, 1139 were the potential or accessible cohort since 637 (35.9%) were lost for Wave II. This group of students was lost for various reasons, such as declining to participate, dropping out from schooling or moving to a different school which was not under study (51.6% were boys and 48.4% girls, and 16.6% had non Spanish nationality). A number of 794 students completed the second measurement and 39 students never returned the written consent form with the guardian’s signature. Therefore, the final sample of 755 (348 boys and 407 girls) represents 42.5% of original cohort and 66.3% of accessible cohort (see Fig 1). Adolescents in the final sample from Wave I presented a mean age of 12.92 (SD 0.89) ranging between 11 and 16 years old and from Wave II a mean age of 16.26 (SD 1.22) ranging between 14 and 19 years of age. Median grade level was 7th grade (44.5%) in Wave I and 10th grade (42%) in Wave II and the following most frequent grade levels were 8th grade (29.4%) in Wave I and 11th grade (27.55%) in Wave II. Participants belonging to high socio-economic family status were 66.8% while 31.4% belonged to low-middle family status. Most participants have Spanish nationality (91.65%) and the remaining ones were foreigners residing in Spain (8.35%).

### Procedure

The fieldwork took place from October to December 2010 in Wave I and from October to December 2013 in Wave II. During these time frames, data were collected in the classrooms by two members of the research team, in absence of the schoolteacher, and the instruments used in this study took around 20 minutes to be filled. A protocol for administering the questionnaire was established. Only participants that returned the written consent form were included in the study.

### Instruments

The Adolescent Sedentary Activity Questionnaire (ASAQ) was employed to assess the time each adolescent spent regularly in daily sedentary behaviors [27]. An intraclass correlation coefficient range of 0.61–0.88 showed an acceptable reliability, similar to the range found in Australian adolescents (0.76–0.90) [27]. A correspondence with accelerometer-determined sedentary time in adolescent girls showed good validity plus test-retest reliability [28]. ASAQ was also used previously in other international studies including Spanish samples [14, 29–33] and was qualified as an acceptable tool by the Lubans et al.’s review [34] about measurement methods of physical activity and sedentary behavior. In the present study a linguistic validation was done by back translation.

ASAQ is a self-report instrument which is organized into three sedentary categories: academic activities (i.e. to study and do the homework with or without a computer); technological-based activities (e.g. watching TV/videos/DVDs and playing with computers, video consoles or mobile phones); and social-based activities (i.e. sitting out with friends and chatting on their computers or mobiles). Variables regarding mobile phone usage were added in order to update the questionnaire.

The academic performance was assessed by requesting to students their academic results from the previous school year to be subsequently grouped in four categories (1 = more than three failed subjects; 2 = between one and three failed subjects; 3 = no failed subjects and average grades; 4 = no failed subjects and high grades). Categories 1 and 2 correspond to students with poor academic achievement and categories 3 and 4 to students with good achievements. Data from academic performance were, therefore, self-reported by students. This instrument has been used in previous studies regarding sedentary activities and academic performance [14, 31].

Several sociodemographic variables were also gathered to accurately describe the participants of this study. Age, gender, socio-economic family status and nationality were data collected from questions directly requested to participants.

### Data Analyses

**Fig 2:**
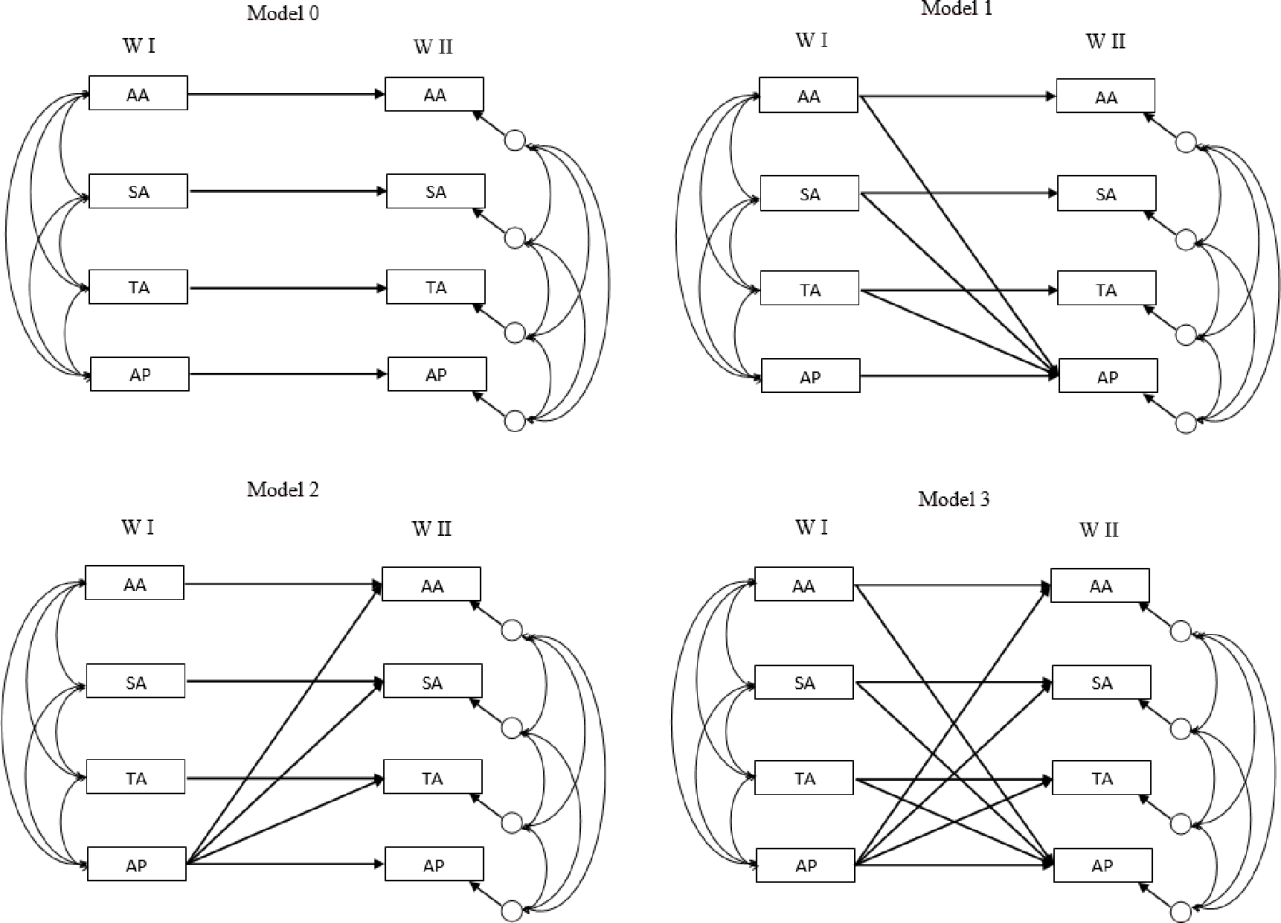
Competing models AA: Academic activities; TA: Technological-based activities; SA: Social-based activities; AP: Academic performance; W I: Wave I; W II: Wave II.

Descriptive statistics were calculated for each variable from the cohort in Waves I and II. To test the fit of the data to the hypothesized models and plausible causal hypothesis, structural equation modeling (SEM) analyses were conducted using EQS (version 6.7). In particular, a comparative analysis was performed to assess four competing models (see Fig 2) and to determine which model best fitted the data. The competing models were:

1. Model 0 (M0). Self-predicted variables or auto-regressive model.
2. Model 1 (M1). Sedentary activities predicted academic performance.
3. Model 2 (M2): Academic performance predicted sedentary activities.
4. Model 3 (M3): Academic performance and sedentary activities predicted each other.

After establishing a best-fitting model, this model was separately tested by gender in a multigroup analysis. A multigroup procedure follows a sequence of steps. As a first step, good model fit should be established for both groups (male and female). After good model fit has been separately established, a configural model is tested. This is a proper multigroup model in which both samples are tested simultaneously, but all parameters are free to be estimated. This model establishes the baseline fit against which to compare more restricted models. Then a fully constrained model was tested. If the fit of this model is the same as the baseline or configural model, then this is evidence of no gender differences in the modeled process. If the fit is deteriorated, then some constraints must be released, those that produce an improvement in fit.

Maximum likelihood (ML) estimation with robust standard errors and *chi square* statistic was employed. The overall fit of the structural models was assessed using different indices with different rationale according to literature [35]. Goodness-of-fit index (GFI) and comparative fit index (CFI), whose minimum value to accept a model is .90, being 1 a value of a perfect fit. Root mean squared error of approximation (RMSEA) and Standard root mean residual (SRMR), whose values between .08-.05 or less would indicate a reasonable amount of error and a value of .05 or below represents an excellent fit. Finally, the parsimony index of each model, and the comparison of them, was assessed using Akaike’s information criterion (AIC) [36]. This index assesses the quality of the fit of the model as a function of the number of coefficients estimated to achieve that level of fit. If two models have very similar AICs, then the most parsimonious model is preferred, since the results are not the product of over-adjusting the data. *chi square* statistic was also informed.

## Results

### Descriptive Statistics

Each sedentary behavior variable had mean scores equal or above 1 hour and 20 minutes of usage per day. In particular, adolescents from Wave I spent a mean of 2.08 ±1.31 hours daily participating in technology-based activities hence becoming the activities in which students spent more time. Social-based leisure activities reached the highest amount of time (3.00 ± 1.88 hours per day) in Wave II. The overall mean score of sedentary behaviors increased by 2 hours and 5 minutes from Wave I (4.80 ± 1.88) to Wave II (6.88 ± 2.27). Finally, academic performance mean values were 3.48 ± 0.86 (Wave I) and 3.21 ± 0.98 (Wave II), indicating good achievements (see Table 1).

**Table 1:** Descriptive statistics for sedentary activities and academic performance.

		Cohort in Wave I					Cohort in Wave II			
Parameter	OSA	AA	TA	SA	AP	OSA	AA	TA	SA	AP
**Mean**	1.60	1.33	2.08	1.38	3.48	2.29	1.82	2.05	3.00	3.21
**SD**	1.16	0.78	1.31	1.15	0.86	1.57	1.22	1.25	1.88	0.98
**Skewness**	1.48	0.77	1.36	1.44	-1.31	1.39	1.19	1.16	0.97	-0.83
**Kurtosis**	3.75	0.71	2.77	3.27	0.84	2.80	3.59	1.92	1.23	-0.37

Time-related values are expressed in decimal notation. OSA: Overall sedentary activities; AA: Academic activities; TA: Technological-based activities; SA: Social-based activities; AP: Academic performance

### Model Comparison

Goodness-of-fit indices for the four competing models are presented in Table 2. Models 1, 2 and 3 fitted the data well. Model 0 (auto-regressive model) had poor fit. Specifically, in M1 to 3, CFI and GFI were above .90, the SRMR values were smaller than .05 and the RMSEA approached the satisfactory fit values, in particular Model 2 with the value being close to .05. CFI did not reach .90 in M0 and SRMR value was over .05. Therefore, M0 was not considered for further comparisons.

**Table 2:** Goodness-of-fit indices for the four competing models.

Goodness-of-fit	Model 0	Model 1	Model 2	Model 3
**χ^2^ robust**	59.14	53.10	33.71	27.54
**Degrees of freedom (df)**	12	9	9	6
**P**	.000	.000	.000	.000
**CFI robust**	.899	.906	.947	.954
**GFI**	.98	.983	.988	.99
**RMSEA robust**	.072	.081	.061	.069
**(90% CI)**	(.05,.09)	(.06, .10)	(.04, .08)	(.04, .10)
**SRMR**	.051	.047	.037	.032
**AIC**	35.14	35.01	15.71	15.54

A *chi-square* contrast test (∆χ^2^) was carried out to determine differences between the three models with a good fit to the data. Considering that two models are different if the *chi-square* difference is significant [37], this test showed that M1 and M3 were different models (∆χ^2^ = 26.54; ∆df = 3; *p* < .001) and M2 and M3 were equivalent (∆χ^2^ = 6.35; ∆df = 3; *p* = .07).

Once it was determined that M2 and M3 had the same statistical fit, and given that the values of the different indices, including the AIC parsimony index, for both models were almost identical (see Table 2), M2 is the model with the larger *df* and fewer parameters, and it is therefore, the most restricted and parsimonious model. According to Hu and Bentler [35], if two mathematical models explain equivalently the reality, the simplest one is chosen since random significant effects are restricted. Therefore, as a result of the model comparison analysis M2 emerged as the best model among the four ones previously hypothesized.

### Portraying the Best Model

**Fig 3:**
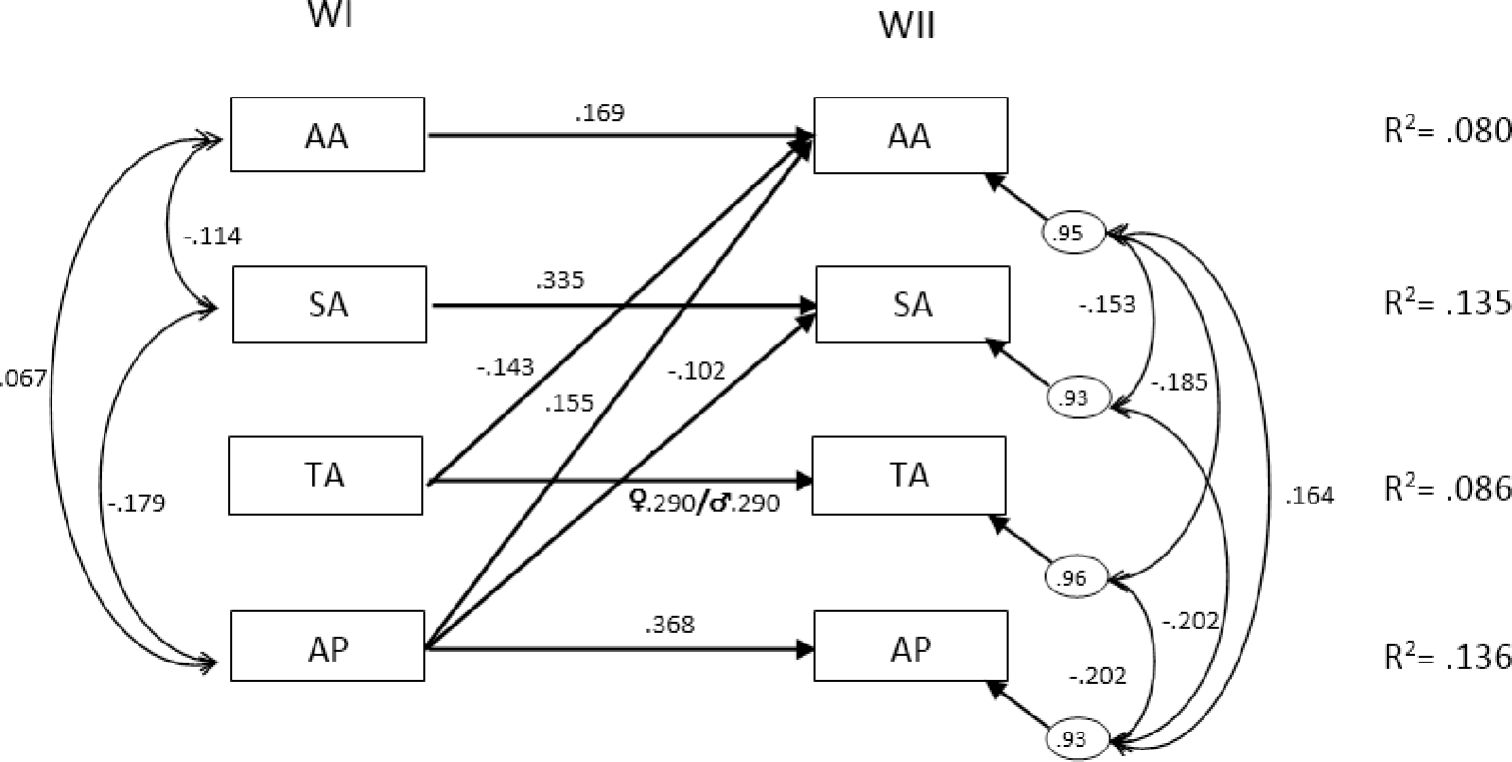
Best Model with correlations and standardized structural effects. Notes: All arrows had significant estimates (*P* < .05); R^2^ = proportion of variance explained; AA = academic activities; SA = social-based activities; TA = technological-based activities; AP = academic performance

The Lagrange Multiplier Test (LM) revealed a relationship between technology-based activities in Wave I and academic activities in Wave II, which also improved the goodness of fit of Model 2 (CFI_robust_ = .979; GFI = .994; RMSEA_robust_ = .041 (90% CI: .02, .07); SRMR = .026). Fig 3 shows the Best Model, which includes covariations among the variables and standardized values. This model indicated that adolescents’ academic performance predicted the time they spent on sedentary activities. It also showed positive auto-regressive effects on the four variables with academic performance and social-based activities being the strongest ones. These two variables accounted for 13% of the variance in academic performance and social-based activities in Wave II. Furthermore, academic performance exhibited a positive longitudinal effect on academic activities and a negative one on social-based activities. Thus, students who performed better academically in early years of adolescence were more likely to spend more time on academic activities and less time on social-based behaviors when they are older. Technological-based activities also showed an inverse longitudinal effect on academic activities. Therefore, students who spent more time on technological-based activities were more probable to devote less time to academic activities three years later.

Examination of the pattern of correlations within Wave I revealed that academic performance had a negative relationship with social-based sedentary activities and this variable had a negative relationship with academic sedentary activities as well. Covariations in Wave II showed more associations among variables than in Wave I. The better the adolescents performed academically, the more time they devoted to academic activities and less to social-based and technology-based activities. Finally, the longer adolescents’ time on social-based and technology-based activities the shorter their time on academic activities.

### Multigroup Model by Gender

The retained model (the one with best fit to the data) was separately tested in girls and boys (see S1 Table, S2 Table, S3 Table), showing an adequate fit in both groups: girls (χ^2^ = 21.78, p = .001, CFI = .96, GFI = .99, RMSEA = .04, SRMR = .05) and boys (χ^2^ = 30.21, p < .001, CFI = .92, GFI = .98, RMSEA = .07, SRMR = .05). Once adequate fit was established separately in both groups, a set of multigroup SEM models was performed, as explained in the ‘Data Analyses’ section: 1) Baseline model (no cross-group constraints; 2) All-constrained model, in which all parameters were constrained to be equal across girls and boys. This tested the hypothesis that there were no differences in the relationships between girls and boys, thus no moderation effects. Model fit for the model with all parameters constrained was deteriorated compared to baseline results (see Table 3). Lagrange Multiplier tests (modification indices) pointed at the need to release the constraint on TA’s auto-regressive effect. After releasing this constraint, this new modified model (Multigroup 3) showed a good fit (see Table 3). This final model suggests that a moderation effect by gender was present in the auto-regressive effect related to the technology-based activities, being higher in boys compared to girls (see this effect in Fig 3).

**Table 3:** Goodness of fit for the hierarchical multigroup models.

Goodness-of-fit	Multigroup 1 (Baseline)	Multigroup 2 (All constrained)	Multigroup 3 (TA’s auto-regressive unconstrained)
**χ^2^ robust**	53.46	69.62	63.21
**Degrees of freedom (df)**	26	34	33
**P**	.000	.000	.000
**CFI robust**	.938	.919	.932
**GFI**	.982	.976	.978
**RMSEA robust**	.053	.053	.049
**(90% CI)**	(.03, .07)	(.03, .07)	(.03, .07)
**SRMR**	.049	.056	.052
**AIC**	1.458	1.622	-2.790

## Discussion

This study compares four hypothesized models of prediction between adolescents’ time spent on different sedentary behaviors (academic activities, technology-based and social-based activities) and academic performance. Our results indicate that the Best Model is the M2 improved once the LM-test was applied. It supports that academic performance is a better predictor of sedentary behaviors in adolescence than the other way round.

According to the Best Model, the positive auto-regressive effects of the four variables under study are consistent with the general ‘success breeds success’ hypothesis supported by the academic achievement field [38], as well as other domains such as information science and technological innovation [39, 40]. In particular, the academic tracking effect of .368, the highest in our study, can be placed between the effects of .430 and .190 found in the two auto-regressive models tested by Jackson et al. [15], but far from .740 and .967, pointed out by Salanova et al. [38] and Ross and Broh [22], respectively. These distances can be explained by the variation in the type of samples used, since the latter were students from 12th grade or undergraduate university students, while the previous samples were students from compulsory schooling, which gathers students with a wider range of academic performance. Even so, the results of this study reveal that students who obtain excellent academic results are more likely to succeed academically in the future and those who spend more time on the three different types of sedentary behaviors in early adolescence are more probable to engage more time in those sedentary behaviors during late adolescence. Although the proportion of variance accounted for from Wave I to Wave II was not large, the importance of the predictive capacity of the type of analysis employed should be stressed.

Furthermore, academic performance also shows a negative longitudinal effect on social-based activities and a positive one on academic behaviors. These relationships indicate that students who perform better academically in early years of adolescence have a higher probability of spending more time on academic activities and less time on social-based behaviors when they are older. Therefore, academic performance in early adolescence may have a motivating force on sedentary behaviors, such as doing homework or studying with and without a computer, while they might have the opposite effect on behaviors, such as sitting out with friends or chatting via social networks in late adolescence. Consequently, a high academic performance may play a protective role in the (excessive time of) future social-leisure behaviors in a similar way as a protective role in the future use of drugs, as revealed in other studies [23, 24].

Technological-based activities also appear as an important predictor in the final model (see Fig 3). They show a negative effect on academic behaviors three years later, and also an increase in the auto-regressive effect moderated by gender. These results are consistent with previous studies that find boys spending more time on overall screen media and technological-based activities than girls [41, 42]. As previously suggested, it may be due to a males’ preference for individual activities, personal challenges or contests with peers, while females prefer social activities for communicating with friends [43]. Nevertheless, these results differ from other studies that show a decrease of time devoted to technological-based activities with the age of adolescents, especially TV and videogames [41, 44]. Moreover, the negative effect of these activities on academic behaviors three years later suggests a displacement hypothesis between both behaviors deferred in time. This hypothesis is found in cross-sectional studies, but it is also observed in longitudinal studies since excessive time spent on technological-based activities is associated with long-term risk of attention difficulties, infrequent homework completion, reducing reading time and poor grades in adolescents and young adults [9, 11, 15, 45, 46]. Therefore, sedentary behaviors linked to screen media usage (playing with video consoles or watching TV) in early adolescence are likely to displace academic behaviors when getting older, probably due to a habit acquired several years earlier.

The significant covariate effects in Waves I and II are similar to most of significant longitudinal effects. It is important to know the amount of relationships in Wave II. The positive association between academic behaviors and academic performance, considering that academic behaviors receive a negative longitudinal effect from technological-based activities in Wave I, are in line with those studies that support the importance of several academic behaviors as mediators between media use and academic performance [11, 15, 45, 46, 47]. On the contrary, the negative relationships of social and technological-based activities with academic performance are similar to the results of other cross-sectional studies on adolescents, some referring to a displacement mechanism [7, 24, 46]. In fact, the need (and pressure) older adolescents in High School (grades 11 and 12) have to get good marks to be able to study their preferred choice of studies in higher education may help to explain the positive link between academic performance and academic activities and the negative association with social-based activities. In any case, these covariate relationships, not only indicate the complexity of different types of sedentary behaviors and academic performance relationships, but also the in-depth search necessity to explain academic performance and sedentary behaviors beyond ‘success breeds success’ and displacement hypotheses. Therefore, this contribution may open new possibilities in addressing future studies regarding the relationships between academic performance and sedentary behaviors.

## Strengths and Limitations

To our knowledge, this is the first study that empirically supports academic performance is a better predictor of sedentary activities than the other way round. This is a timely topic addressed using a longitudinal study and multiple types of sedentary behaviors, as well as methods coherent with the questions asked. However, there are some limitations that should be considered when interpreting the results from this study. The use of retrospective self-reported data by ASAQ to assess sedentary activities may raise some concern about the reliability and accuracy of the data. Nevertheless, it has been previously proven reliable and valid [26, 34] and used in several studies from different countries [14, 29, 30]. Also, measurement error was minimized through employing standardized protocols and guidelines, especially data from mobile phone questions that were added to the ASAQ following the same fieldwork protocol. Finally, the possible longitudinal effects of other variables should be hypothesized (e.g. gender, family socioeconomic status, peers’ acceptance/rejection and/or school ethos) because they plausibly affect the current relationships. This would require testing new models that include potential mediators and studying more time points in future investigations.

## Conclusions

This study examines whether the time secondary school adolescents spend on different types of sedentary behaviors is a better predictor of academic performance than reverse and whether a moderating effect by gender exists. Results indicate it is one of the first studies which suggests, based on empirical evidence, academic performance predicts time spent on sedentary behaviors in adolescence. The study shows positive auto-regressive effects on the four variables under study with academic performance and social-based activities the strongest ones, thus reinforcing the ‘success breeds success’ hypothesis. Among the significant longitudinal effects, academic performance leads positively over spending time on academic behavior and negatively predicts social-based activities. Furthermore, technological-based activities also showed a moderating effect by gender and a negative longitudinal association with academic sedentary activities that supports a ‘displacement hypothesis’. Longitudinal and covariate effects in this study reflect the complex relationships between sedentary behaviors and academic performance and the need to explore these relationships in depth.

## Implications

According to the results of this study, practical implications are based on the protective role that academic performance may play in future sedentary behavior. In particular, the supportive school community environment for promoting academic success emerges as a salient potential implication. This strategy may become more common among schools, families and communities and easier to be developed in comparison with an environment focused on controlling and reducing screen-related sedentary use among school adolescents. Some rationales and strategies, emerging from literature on youth risk and centered on promoting academic performance [48–51], may be of help since students with poor academic performance are the ones with more screen-related sedentary use. Moreover, fostering good academic performance among younger adolescents may have a reducing effect on social-based sedentary activities in older adolescents and a supporting effect on academic sedentary activities, which are more productive academically, due to the negative longitudinal effects of academic performance on social-based activities identified in our study. The specific roles schools play for improving academic performance should focus on teaching methodologies with screen media use as part of their teaching-learning processes. If mobile phones and tablets are included in the development of academic activities and are useful for adolescents’ academic performance, as previously happened with the usage of calculators in classrooms, they could literate students in a technological responsible and moderate use of screen media beyond the school.

## Supporting Information

S1 Table Best Model covariance matrix for the whole sample. (DOCX)

S2 Table Best Model covariance matrix for boys. (DOCX)

S3 Table Best Model covariance matrix for girls. (DOCX)
