# Correction: Does Sedentary Behavior Predict Academic Performance in Adolescents or the Other Way Round? A Longitudinal Path Analysis

**DOI:** 10.1371/journal.pone.0158254

**Published:** 2016-06-22

**Authors:** Jorge Lizandra, José Devís-Devís, Esther Pérez-Gimeno, Alexandra Valencia-Peris, Carmen Peiró-Velert

There is an error in Fig 3 in the moderation effect by gender in the autoregressive effect related to the technological-based activities (TA). Instead of the values “♀ .290/♂ .290”, the correct values are “♀ .141/♂ .323”. Please see the corrected Fig 3 here.

**Fig 3:**
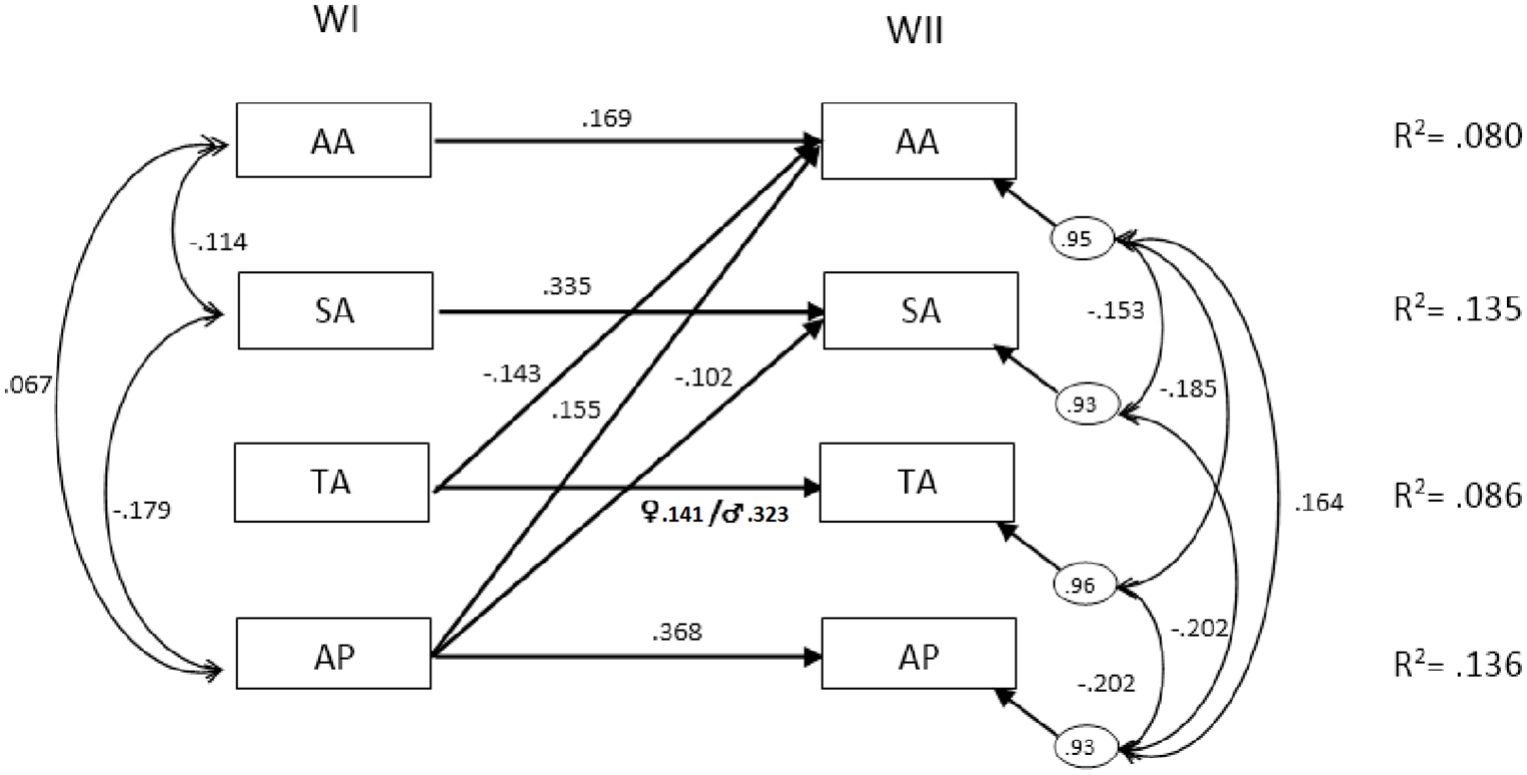
Best Model with correlations and standardized structural effects. Notes: All arrows had significant estimates (P < .05); R2 = proportion of variance explained; AA = academic activities; SA = social-based activities; TA = technological-based activities; AP = academic performance.
