# Supplementary material for: Does Sedentary Behavior Predict Academic Performance in Adolescents or the Other Way Round? A Longitudinal Path Analysis
Source: PLoS One. 2016 Apr 7;11(4):e0153272. doi: 10.1371/journal.pone.0153272 (PMC4824448; doi:10.1371/journal.pone.0153272)
Supplement: S1 Table — (DOCX) [file pone.0153272.s001.docx]

**S1 Table. Best Model covariance matrix for the whole sample.**

|  | AA1 | SA1 | TA1 | AP1 | AA2 | SA2 | TA2 | AP2 |
| --- | --- | --- | --- | --- | --- | --- | --- | --- |
| AA1 | .612 |  |  |  |  |  |  |  |
| SA1 | -.103 | 1.334 |  |  |  |  |  |  |
| TA1 | -.047 | .102 | 1.702 |  |  |  |  |  |
| AP1 | .045 | -.178 | -.029 | .746 |  |  |  |  |
| AA2 | .195 | .019 | -.256 | .179 | 1.505 |  |  |  |
| SA2 | -.142 | .762 | -.042 | -.263 | -.349 | 3.523 |  |  |
| TA2 | -.038 | -.005 | .535 | -.058 | -.349 | -.031 | 1.558 |  |
| AP2 | .069 | -.108 | -.109 | .312 | .276 | -.450 | -.268 | .961 |
