# Supplementary material for: Does Sedentary Behavior Predict Academic Performance in Adolescents or the Other Way Round? A Longitudinal Path Analysis
Source: PLoS One. 2016 Apr 7;11(4):e0153272. doi: 10.1371/journal.pone.0153272 (PMC4824448; doi:10.1371/journal.pone.0153272)
Supplement: S2 Table — (DOCX) [file pone.0153272.s002.docx]

**S2 Table. Best Model covariance matrix for boys.**

|  | AA1 | SA1 | TA1 | AP1 | AA2 | SA2 | TA2 | AP2 |
| --- | --- | --- | --- | --- | --- | --- | --- | --- |
| AA1 | .655 |  |  |  |  |  |  |  |
| SA1 | -.142 | 1.104 |  |  |  |  |  |  |
| TA1 | -.067 | .280 | 1.923 |  |  |  |  |  |
| AP1 | .022 | -.184 | -.040 | .812 |  |  |  |  |
| AA2 | .194 | -.101 | -.214 | .187 | 1.107 |  |  |  |
| SA2 | -.175 | .555 | -.051 | -.300 | -.254 | 3.009 |  |  |
| TA2 | -.010 | .046 | .642 | .022 | -.325 | -.134 | 1.994 |  |
| AP2 | .064 | -.238 | -.032 | .271 | .257 | -.530 | -.234 | .959 |
