# Supplementary material for: Does Sedentary Behavior Predict Academic Performance in Adolescents or the Other Way Round? A Longitudinal Path Analysis
Source: PLoS One. 2016 Apr 7;11(4):e0153272. doi: 10.1371/journal.pone.0153272 (PMC4824448; doi:10.1371/journal.pone.0153272)
Supplement: S3 Table — (DOCX) [file pone.0153272.s003.docx]

**S3 Table. Best Model covariance matrix for girls.**

|  | AA1 | SA1 | TA1 | AP1 | AA2 | SA2 | TA2 | AP2 |
| --- | --- | --- | --- | --- | --- | --- | --- | --- |
| AA1 | .566 |  |  |  |  |  |  |  |
| SA1 | -.094 | 1.483 |  |  |  |  |  |  |
| TA1 | .014 | .039 | 1.364 |  |  |  |  |  |
| AP1 | .061 | -.181 | -.007 | .690 |  |  |  |  |
| AA2 | .152 | .030 | -.132 | .159 | 1.680 |  |  |  |
| SA2 | -.160 | .848 | .128 | -.246 | -.599 | 3.978 |  |  |
| TA2 | .004 | .085 | .209 | -.105 | -.124 | .305 | 0.828 |  |
| AP2 | .057 | -.031 | -.116 | .342 | .233 | -.443 | -.207 | .942 |
